# Non-Invasive Skin Cancer Diagnosis by Electrical Impedance Spectroscopy: Biophysics, Devices, Clinical Evidence, and Future Directions

**DOI:** 10.3390/diagnostics16162673

**Published:** 2026-08-21

**Authors:** Jing Yang, Ling Wu, Huan Xue, Jingxiu Chai, Yuchong Chen, Cheng Zhong

**Affiliations:** 1 Department of Dermatologic Surgery, Shanghai Skin Disease Hospital, School of Medicine, Tongji University, Shanghai 200443, China; 2602045@tongji.edu.cn (J.Y.); 2602118@tongji.edu.cn (L.W.); 2602046@tongji.edu.cn (H.X.); 2602117@tongji.edu.cn (J.C.); 2 Tianjin Key Laboratory of Composite and Functional Materials, School of Materials Science and Engineering, Tianjin University, Tianjin 300072, China

**Keywords:** electrical impedance spectroscopy, bioimpedance, skin cancer, melanoma, non-invasive diagnosis, dermoscopy, biosensors, medical devices

## Abstract

Skin cancer represents a growing global health burden. Current diagnostic pathways combine clinical examination and dermoscopy with histopathological confirmation; however, overlap between benign and malignant lesions can create diagnostic uncertainty and lead to potentially avoidable biopsies. Electrical impedance spectroscopy (EIS) has emerged as a non-invasive technique with potential for portable and cost-efficient implementation that quantifies the dielectric contrast between malignant and healthy tissue, providing objective information that may support clinical decision-making. This review synthesizes the field across four levels. First, we describe the biophysical origins of the impedance contrast in skin cancer, spanning the cellular, tissue architecture, and molecular scales, together with the equivalent circuit and Cole–Cole frameworks used to interpret it. Second, we examine hardware advances, including electrode–skin interface strategies, flexible and wearable architectures, computational electrode design, and the translation from laboratory prototypes to commercial systems such as Nevisense. Third, we critically appraise clinical evidence from large multicenter trials, focusing on the sensitivity–specificity trade-off and the demonstrated reduction in the number needed to excise. Finally, we discuss emerging frontiers, including artificial intelligence-driven analysis and multimodal fusion with dermoscopy, reflectance confocal microscopy, optical coherence tomography, and near-infrared spectroscopy. We conclude that EIS is most valuable as a complementary component within an integrated, AI-supported multimodal diagnostic framework.

## 1. Introduction

With the growing incidence of skin cancer, which has become a global public health concern [1,2], more precise and objective tools are urgently needed to address the inherent limitations of current diagnostic methods [3]. At present, the clinical diagnosis of skin diseases usually first relies on visual observation assisted by dermoscopy, and invasive biopsy follows to offer further confirmation [4,5,6]. Visual observation is highly dependent on the clinical experience of the doctor, since the morphology of early or atypical melanoma is similar to that of benign nevi [4,7]. As a result, considerable numbers of biopsies are performed on benign lesions to avoid missed diagnosis [7,8]. This not only increases the healthcare cost for patients but also exposes them to unnecessary surgery and the accompanying pain and anxiety [7,9].

To address these challenges in early skin cancer detection, a series of non-invasive detection methods have been developed to objectively detect suspicious skin features [7,10,11]. A major example is detection technologies based on optical information. For instance, reflectance confocal microscopy (RCM) can provide high-cellular-resolution imaging of tissues without incisions [12,13,14], and optical coherence tomography (OCT) allows a deeper evaluable depth for the observation of skin cancer [13,14]. Meanwhile, multispectral imaging techniques diagnose suspicious skin lesions through the analysis of distinct signal responses to various wavelengths of light [14]. These advanced techniques are dedicated to providing quantifiable data that could minimize the impact of subjective experience on diagnosis and reduce the incidence of unnecessary biopsies [11,14].

However, a translational gap exists between the clinical promise and the feasibility of these advancements. The most prominent obstacle lies in economic aspects, including the high equipment costs of high-precision systems such as RCM and OCT and the human resources required to operate such equipment [10]. Moreover, the bulky and non-portable nature of these devices [15] further constrains the application scenarios to specialized academic settings and large medical centers [10]. They fail to meet the demand for an objective and efficient tool to guide biopsy decisions, especially in primary care centers such as community hospitals. These centers, where both experienced doctors and advanced testing equipment are lacking, are exactly the places that require such a tool the most [10,16,17].

In response to these limitations, electrical impedance spectroscopy (EIS) has emerged as a promising non-invasive technology with potential for portable and cost-efficient implementation [18,19]. This technique is based on the principle that the tissue’s electrical properties exhibit significant changes during carcinogenesis, which would be reflected in the impedance of the tissue [20]. Moreover, the whole measurement process is painless, with only a low-voltage electrical current applied to the skin lesion, and the changes in the tissue’s composition can be observed [20,21]. The different impedance spectra between cancerous and healthy tissue could serve as quantitative and objective data for the detection of skin cancer [20,22], reducing the impact of subjective interpretation associated with visual observation. Moreover, the underlying principles are conducive to the development of portable and cost-efficient devices [18,19]. As a result, EIS has been investigated as an adjunctive diagnostic tool in primary care settings [23,24].

**Figure 1:**
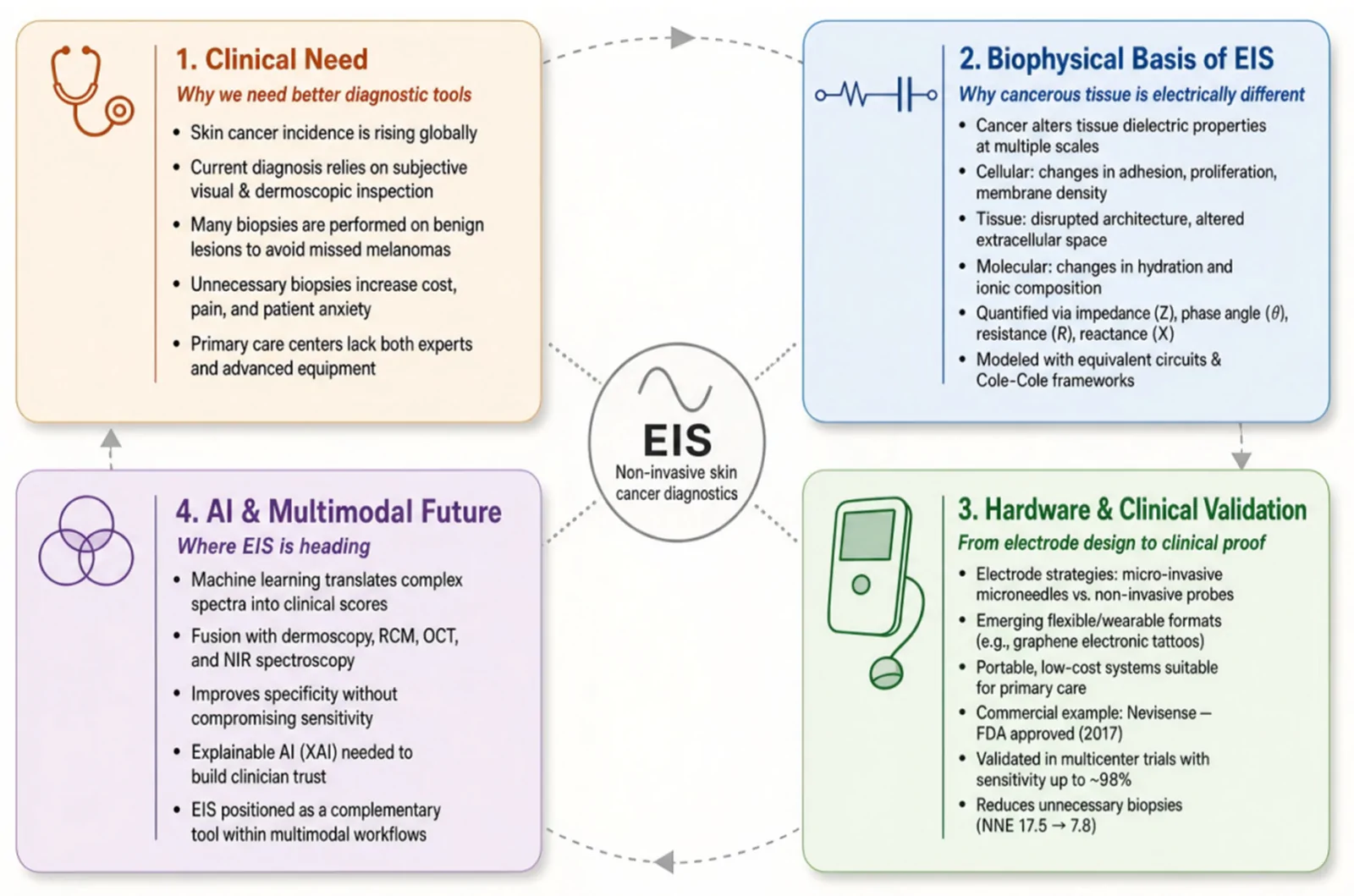
Overview of electrical impedance spectroscopy (EIS) for non-invasive skin cancer diagnosis.

This article provides a comprehensive resource for researchers and doctors during the application of EIS in skin cancer diagnostics (Figure 1). By integrating biophysical mechanisms, device development, clinical evidence, and multimodal strategies, this review addresses the gap between the technical promise and the clinical translation of EIS. The review first discusses the origins of the dielectric contrast between healthy and cancerous tissue and then presents the development of EIS hardware, including the design of electrodes and commercial equipment. In addition, the clinical practice of EIS is collectively evaluated by presenting large-scale experiments involving diagnostic accuracy. Moreover, the review discusses the integration of EIS with advanced artificial intelligence and other non-invasive technologies. Through this review, this paper aims to identify the gaps between labs and clinical scenarios, supporting the development of EIS as a complementary diagnostic tool for skin cancer.

## 2. Biophysical Basis of Bioimpedance Spectroscopy

### 2.1. Origins of Dielectric Contrast in Skin Cancer

#### 2.1.1. The Core Diagnostic Principle: Macroscopic Tissue Alterations

The diagnosis of skin cancer via EIS is based on alterations in the tissue’s electrical properties, especially the dielectric contrast, during disease progression [22]. The dielectric contrast between cancerous and benign tissue has been widely found in different cancers, including pulmonary nodules and head and neck, breast, and skin cancers [25,26,27,28]. For instance, an in vitro analysis of 252 human pulmonary tissues demonstrated that squamous cell carcinomas (SCCs) had higher conductivity and relative permittivity than normal tissues from a frequency range of 100 Hz to 100 MHz. Moreover, adenocarcinomas (ADCs) had higher conductivity at frequencies above 10 kHz [25].

**Figure 2:**
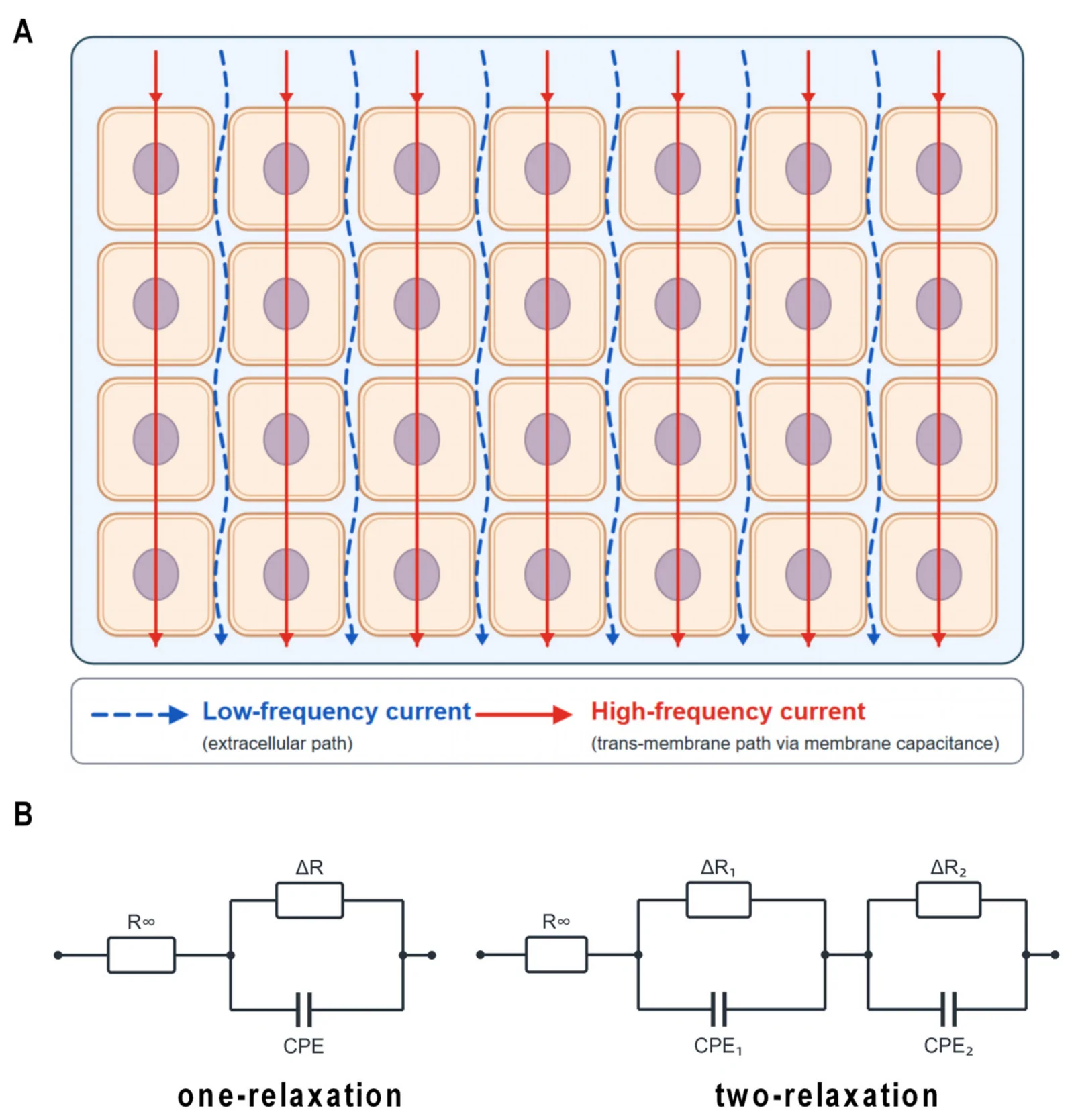
(**A**) Frequency-dependent current pathways in tissue: at low frequencies, the current is largely confined to the extracellular space, whereas, at high frequencies, it also crosses the cell membranes. (**B**) Tissue-level equivalent circuit models based on the Cole–Cole electrical impedance characteristic equation, showing one- and two-relaxation models used to interpret complex macroscopic bioimpedance data.

It should be noted that EIS measurement can assess either local cancerous tissues [29] or broader body regions of patients [27]. Cancerous tissues typically have higher conductivity than healthy tissues [29]. For example, the impedance of cancerous tissues in oral squamous cell carcinoma (OSCC) dropped from 15.4 kΩ in healthy mucosa to 4.4 kΩ in OSCC at 20 Hz [29]. At such a low frequency, the current cannot penetrate capacitive cell membranes but travels through extracellular pathways (Figure 2A). Concurrently, OSCC can enhance the extracellular pathway, therefore reducing the measured impedance [29]. In contrast, if the measurement is performed on the body, such as via hand-to-foot tetrapolar configurations [27], the current passes through the body rather than solely the cancerous tissue. In these cases, patients with breast or head and neck cancers exhibit higher impedance due to cancer-induced malnutrition and muscle wasting [27,28].

#### 2.1.2. Multiscale Biophysical Origins of Electrical Changes

The electrical alterations of cancerous tissues are the accumulation of changes across the cellular, tissue architecture, and molecular scales.

At the cellular scale, the dielectric contrast primarily originates from cell behaviors including cell adhesion, cell proliferation, and cell migration [30]. An in vitro study by Zhang et al. performed EIS measurements on skin cancer cells (A431) and healthy keratinocytes (HaCaT) at frequencies from 1 Hz to 100 kHz. During the monitoring period of five days, A431 cells showed lower normalized impedance than HaCaT cells, and the largest dielectric contrast was at the frequency of 1465 Hz [30]. The lower impedance of cancer cells during proliferation is driven by cell behavior; they tend to aggregate but not form a uniform layer to cover the electrode surface, thus reducing the insulating barrier area [30].

At the tissue architecture scale, the growth of the tumor can disrupt the tissue and thus affect its electrical response [31]. For instance, EIS measurement in head and neck squamous cell carcinoma (H&NC) revealed that the reactance (Xc) dropped from 51.75 Ω in healthy tissues to 47.54 Ω in tumors (*p* = 0.03) at a frequency of 50 kHz [31]. At this frequency, the Xc is mainly determined by the capacitive properties of cell membranes, and its reduction reflects the loss of cell membranes.

At the molecular scale, the electrical properties of cancerous tissues are affected by tissue hydration and the molecular composition [31]. In the aforementioned H&NC study, beyond the drop in reactance, the resistance (R) at 50 kHz increased from 538.58 Ω to 590.13 Ω (*p* = 0.04) [31]. The growing resistance is attributed to the decreased water content in tissues.

### 2.2. The Stratum Corneum as the Primary Measurement Barrier

As the outermost layer of the skin, the stratum corneum (SC) is highly resistive. It can have 350-times higher parasitic impedance than the underlying tissues [32], and this high parasitic impedance can reduce the resolutions of the signals from tissues or tumors deep inside the skin [33]. Furthermore, a dehydrated or dry SC causes additional contact impedance, which further masks the subtle electrical changes of tumors [34].

Interestingly, a specific set of skin diseases can be diagnosed exactly due to alterations in the stratum corneum [35]. For example, cancerous melanoma (MM) damaged skin barrier integrity, reducing the local electrical resistance by half compared to the adjacent healthy tissue [35]. Beyond the static barrier of the SC, the electrical properties of the skin are dynamically affected by systemic physiological factors [22]. Some common physiological factors, including allergies, sweating, and even age and gender, can cause fluctuations in skin impedance [22]. As a result, these inherent physiological differences must be taken into consideration during diagnosis. Rather than viewing these fluctuations as noise, doctors are able to deliberately introduce these physiological differences to acquire clinically useful electrical signals [36]. For example, it has been reported that early-stage and late-stage tumors show different electrical responses to hypoxia under vascular occlusion tests. This result is helpful for determining the stage of cancer progression [36].

### 2.3. Equivalent Circuits and Mathematical Frameworks

EIS measurements of biological tissues generate results in terms of impedance. However, impedance is a complicated type of data consisting of physical parameters including the modulus (Z), phase angle (θ), real part (R), and imaginary part (X), each representing distinct tissue properties [29,37]. For instance, a series of studies on larynx, oral cavity, and tongue cancers showed that cancerous tissues exhibited much lower impedance and resistance [29,37,38]. This trend, however, is not universal across all types of cancer. Cancerous tissues in breast and head and neck cancers exhibited higher resistance than their healthy counterparts [27,28]. The phase angle is another highly sensitive but variable biomarker for the diagnosis of cancer. Research reports that the phase angle has a lower value in specific types of head and neck cancer [28] but a higher value in oral squamous cell carcinoma [29].

It should be noted that all these physical parameters in impedance spectroscopy are frequency-dependent. Therefore, mathematical frameworks and equivalent circuits have been adopted to evaluate cancerous tissues’ electrical responses. At the cellular level, an equivalent circuit model could decouple the impedance into the cell-layer resistance (Rc) and capacitance (Cc). The true value of this mathematical decoupling lies in the ability to extract different dimensions of cellular information simultaneously. For instance, referring back to the proliferation of A431 cancer cells [30], isolating Rc revealed qualitative differences in cell morphology and adhesion. Because A431 cells tend to aggregate, a larger area of the electrode was exposed and thus A431 cells had a lower Rc increase (7.5%) than healthy HaCaT cells (12.9%). In parallel, isolating the Cc values of both A431 and HaCaT cells indicated a linear decrease with the cell number. Therefore, through decoupling the overall impedance, the behavioral characteristics of cancer cells and their proliferation can be quantified via Rc and Cc, respectively.

At the macroscopic tissue level, the bioimpedance of tissues typically exhibits multiple relaxation mechanisms, which can be quantified using a two-relaxation Cole–Cole model (Figure 2B). The α-dispersion band (typically below 1 kHz) is predominantly governed by the extracellular ionic environment. For example, the α-band electrical responses of adenocarcinomas (ADCs) and benign lung nodules are similar, since the sialic acid mucus in ADC and inflammatory exudates in benign lesions would both alter the local ionic concentrations [25]. Conversely, the β-dispersion band (1 kHz to 100 MHz) reflects the capacitance of cell membranes (Cm). Because cancerous tumors possess a higher density of cell membranes due to proliferation, their β-band parameters differ significantly from those of healthy tissues. In pulmonary nodules, the high-frequency limit resistance (R∞) of cancerous nodules is at least one order of magnitude lower than that of normal tissue [25].

While the two-relaxation Cole–Cole model effectively evaluates these frequency-dependent signals, it assumes that tissues are homogeneous and isotropic [39]. However, real tumors could be highly heterogeneous, and the assumption of the two-relaxation Cole–Cole model could introduce measurement errors [39]. To address this limitation, a multilayer and anisotropic model that incorporates conductivity tensors to account for cellular orientation and fiber alignment was developed to reduce measurement errors in heterogeneous tissues by 20–30% [39]. Furthermore, Bello et al. evaluated relaxation dynamics in both the frequency and time domains by applying the Kohlrausch–Williams–Watts (KWW) function to time-domain data. This approach quantified the condition of charge carriers, revealing the increased membrane capacitance and conductivity of cancerous tissue in the clinical validation of 417 cases [40].

## 3. Systems and Hardware for Bioimpedance Spectroscopy

### 3.1. Design of Electrodes, Sensors, and Electronics

#### 3.1.1. Electrode–Skin Interface Strategies: Physical Penetration Versus Electronic Compensation

**Figure 3:**
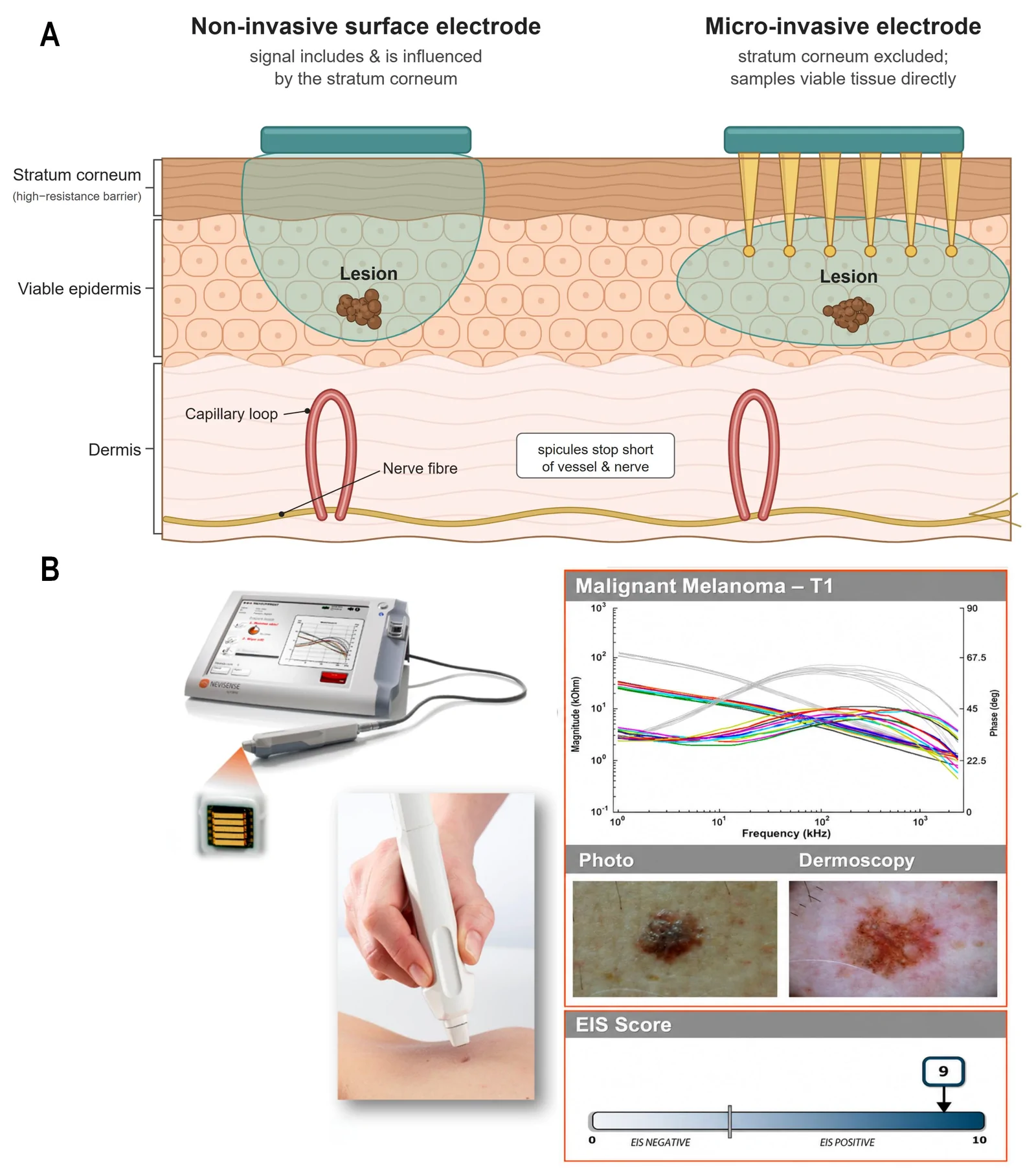
(**A**) The highly resistive stratum corneum as the principal measurement barrier, contrasting a non-invasive surface electrode (current largely blocked at the stratum corneum) with a microinvasive electrode penetrating the stratum corneum into the viable epidermis, without reaching dermal vessels or nerves. (**B**) The Nevisense EIS system in clinical use, showing the device (handheld probe and monitor), the measurement technique on a lesion, the resulting impedance spectrum, and the final risk score. Reproduced from Ref. [10], 2022, MDPI; licensed under CC BY 4.0.

The clinical application of bioimpedance spectroscopy in diagnosis requires the development of elaborate hardware systems. The core challenge in realizing clinical devices lies in the skin’s stratum corneum. As discussed above, the stratum corneum has high impedance, which hinders the acquisition of a clear electrical response. To address this challenge, minimally invasive penetration and electronically neutralizing its contribution through circuit design have been developed as two major technologies (Figure 3A) [32,41].

The microinvasive strategy is based on microneedles that physically penetrate the skin’s SC while not reaching the blood vessels or nerves in the dermis. A commercialized example is the Nevisense system (SciBase AB, Stockholm, Sweden) (Figure 3B) [42]. Its disposable electrode is equipped with triangular gold-covered spicules that are approximately 150 μm high with a 170 μm base. These spicules penetrate the SC to capture the resistive properties of the intracellular environment and the capacitive properties (reactance) of the cell membranes, which are crucial for identifying cancerous cells [42].

Historically, the choice between microinvasive and non-invasive electrodes was strictly dictated by the depth of the target lesion. As early as 2005, Åberg et al. demonstrated that conventional non-invasive probes were superior for detecting basal cell carcinomas (BCCs). In contrast, microinvasive electrodes exhibited higher sensitivity and specificity in detecting cancerous MM [41].

However, recent advancements in circuit design have alleviated the hardware limitations of non-invasive electrodes. For example, a four-wire (tetrapolar) non-invasive system can measure the impedance of deep tissues under the interference of a highly resistive SC [32]. This non-invasive system employed active shielding for the sensing electrodes and passive shielding for the force electrodes, reducing the parasitic capacitance errors seen in early systems [32].

#### 3.1.2. Electrode Materials, Mechanical Properties, and Flexible/Wearable Architectures

Alongside the strategy that addresses the SC barrier, the choice of electrode material and its physical form directly affect signal quality and measurement stability. Conventional electrodes, including Ag/AgCl electrodes, gold-plated electrodes, and electrical muscle stimulation (EMS) electrodes, have various advantages and disadvantages. For instance, gold-plated electrodes achieved a higher signal-to-noise ratio (SNR) of 30 for chest-based respiratory measurements, compared with 10 for Ag/AgCl and 15 for EMS electrodes. In contrast, Ag/AgCl showed the lowest chip error rate (CER) of 3.30% in body-coupled data transmission, outperforming gold-plated electrodes (5.50%) [43]. This study reveals that there is no universally superior material, and the selection of electrode materials should be driven by the specific diagnostic requirements [43].

A limitation that is shared by rigid electrodes is that they can cause local skin deformation during measurement, which introduces temporal impedance artifacts that reflect mechanical stress rather than tissue properties [44]. This limitation has led to great research interest in novel flexible materials, which allow stable and continuous long-term measurement. For instance, soft and conformable epidermal devices can be directly attached to the skin as dry electrodes, mitigating the high parasitic contact impedances without the need for intermediate gels [32]. Among these flexible solutions, graphene electronic tattoos (GETs) have been considered as a promising alternative [45,46]. GET arrays feature an ultrathin, flexible design that ensures conformal contact with the skin. Multi-electrode GETs can be engineered into specific shapes to suit stretchable skin and allow for the precise localization of target areas [45]. These bilayer graphene structures have the advantages of biocompatibility, a lightweight design, transparency, and sweat permeability, which make them suitable for wearable sensors [46].

#### 3.1.3. Array Geometry and Computational Design for Depth-Resolved Sensing

Beyond the material and physical form of single electrodes, the spatial arrangement of electrodes introduced a third design principle that governs the electric field’s penetration depth and measurement sensitivity. Multifrequency electrical impedance has been investigated as a screening tool for skin cancer identification [47]. Among various computational tools, the finite element method (FEM) has emerged as a powerful approach that can evaluate the geometric relationships of electrode arrays by simulating current distribution [48,49]. By extracting precise cellular and geometrical parameters from histological sections of normal skin, researchers can construct highly accurate multilayer skin models. However, when meshing the thin structures in these detailed skin tissue models, the number of computational cells increases considerably. This significantly extends the computational time and thus limits the maximum complexity of the model [48]. In order to address this computational bottleneck, researchers have modeled lipid bilayers with dielectric surfaces [48]. One model showed that microinvasive electrodes separated by 1 mm reached only the papillary dermis, while extending the separation to 6 mm drove the current approximately 2.5 mm into the reticular dermis [48]. In addition, these FEM models can demonstrate how the current density distribution varies with the frequency and tissue health. For instance, simulations reveal that, at a low frequency of 500 Hz, an in situ melanoma provides an increased conductivity pathway compared to normal skin, whereas, at 1 MHz, the differences become more subtle as cell membranes are short-circuited and the current flows through deeper layers [48]. A study on a tetrapolar probe also reported the influence of the electrode diameter and separation distance on the sensitivity [50]. FEM simulations showed that, for a 1 mm^2^ epidermal tumor, impedance measurement achieved only ~1% sensitivity, whereas a tetrapolar configuration with a 1 mm electrode diameter and 1.8 mm separation achieved ~8% [50].

#### 3.1.4. Microfabricated Biosensor Platforms for In Vitro Analysis

Bioimpedance technology was originally designed for in vivo tissue imaging, and researchers are extending its use to in vitro cellular testing. To enhance the efficiency of such in vitro research, high-throughput screening is essential. Microfabrication technology can be used to develop integrated sensor platforms that enable precise impedance analysis in biological models such as individual cells [51,52].

For example, in single-cell research, scientists utilized photolithography to develop microdevices for the impedance analysis of cancer cells [52]. It was revealed that B16-F10 cancer cells had an average specific membrane capacitance of 1.154 ± 0.29 μF cm^−2^ and membrane resistance of 3.9 ± 1.15 kΩ cm^−2^ [52]. Complementing this characterization, an automatic mathematical modeling method for multielectrode impedance measurements was developed and validated on B16-F10 melanoma cells over a 24 h period, achieving up to 97% accuracy in impedance assessment while operating on minimal computational resources such as an 8-bit microcontroller [51].

### 3.2. Development of Portable and Integrated Diagnostic Systems

#### 3.2.1. Clinical Scenario-Driven Design Requirements: Portability, Usability, and Cost

The development of EIS devices for skin cancer diagnosis is in line with the demands of clinical scenarios. Primary care centers represent the first line for skin cancer detection. However, a recent clinical study involving 61 primary care providers (PCPs) demonstrated that visual evaluation showed low sensitivity of 69.2% and specificity of 44.0% in detecting melanomas and severely dysplastic nevi [23]. To address the diagnostic challenges in primary care, there is an urgent demand for objective diagnostic devices with portability, affordability, and ease of use [18,19].

In terms of hardware, low-cost microcontrollers and System-on-Chip (SoC) designs have attracted great research interest, promising to replace the traditional bulky equipment. For example, Khusnul et al. developed an Arduino-based EIS system working at a frequency of 5 Hz to 200 kHz with a current of 0.370 mA [19]. Similarly, Lloyd et al. developed a handheld EIS system, enabling handheld probes to collect and transmit data without limiting the movement of clinicians [53].

Beyond hardware design, the data processing of EIS systems should also be specifically designed for clinical usability. Raw impedance data comprise complex components, including the phase angle and reactance, which are almost uninterpretable among non-specialists. Automated algorithms are urgently required to translate impedance data into intuitive metrics, such as an EIS score indicating the skin cancer likelihood [23].

#### 3.2.2. From Lab Prototypes to Commercial Devices: The Case of Nevisense

To meet the clinical demands for portability and usability, the Nevisense system has emerged, becoming the leading commercialized EIS device. The Nevisense system consists of a portable touch-screen monitor connected to a handheld probe with disposable electrodes [42]. The disposable nature of the electrodes ensures a hygienic detection process, while the handheld probe facilitates measurements on different regions of patients’ bodies. The detection protocol is also specifically designed. Firstly, saline is applied to the skin to standardize its hydration, followed by measurements on healthy skin adjacent to the suspicious lesion to establish a referential baseline. Subsequently, measurement is performed on the lesion, which takes a short time, within 10 s [42].

**Figure 4:**
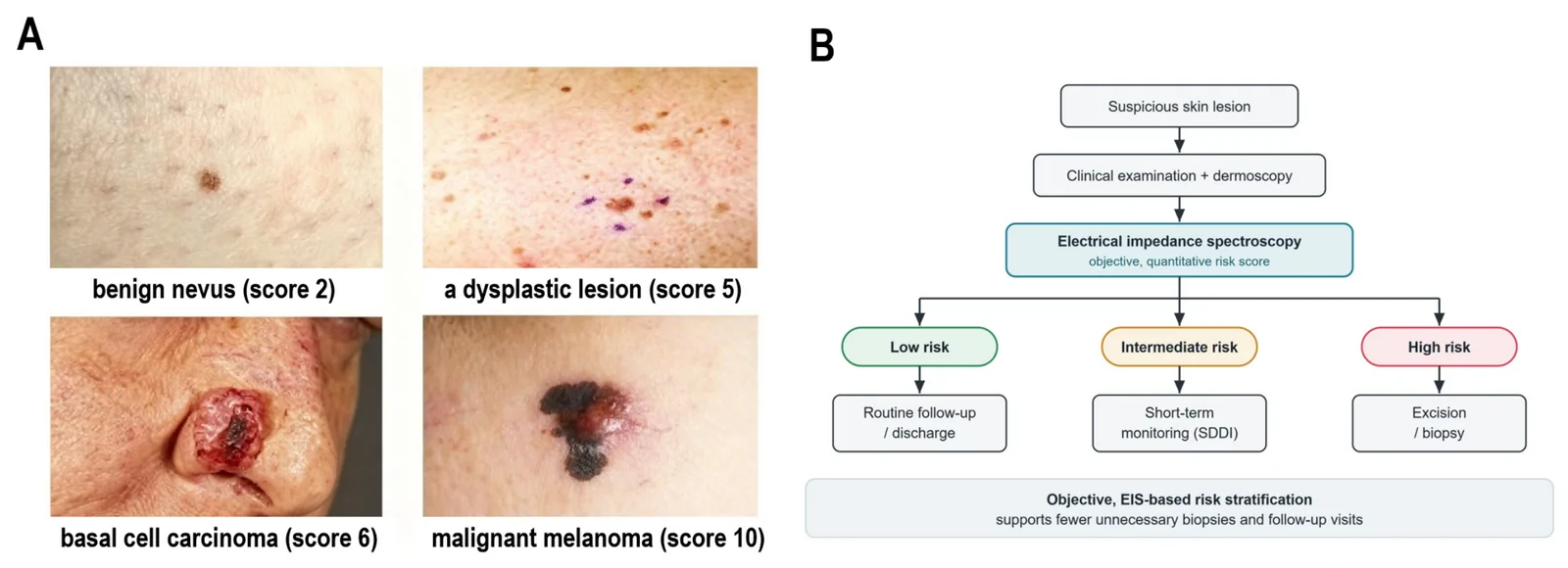
(**A**) Representative macroscopic images of skin lesions with corresponding Neviscores, including a benign nevus with score 2, a dysplastic lesion with score 5, a basal cell carcinoma with score 6, and a malignant melanoma with score 10. Reproduced from Ref. [54], 2022, MDPI; licensed under CC BY 4.0. (**B**) Conceptual schematic of how EIS functions as an objective triage step within the diagnostic workflow: an EIS-derived risk score stratifies lesions into low-, intermediate-, and high-risk groups to guide routine follow-up/discharge, short-term sequential digital dermoscopic imaging (SDDI) monitoring, or excision/biopsy, respectively.

Moreover, the device has an internal algorithm that processes the measured impedance data into a “Neviscore” ranging from 0 to 10, which reflects the malignancy of the lesion (the higher the value, the greater the malignancy). As shown in Figure 4A, this scoring system has been applied to different skin lesions, yielding a low score of 2 for a benign nevus and a score of 5 for a dysplastic lesion, as well as higher scores for BCC (score 6) and MM (score 10) [54].

Despite its successful commercialization, there is still room to optimize the Nevisense system. This system has a rigid electrode with an approximately 5 × 5 mm active area, hindering measurement on lesions smaller than 2 mm or larger than 20 mm [55]. Future commercial devices could introduce sensor arrays that can flexibly adjust the detection area.

#### 3.2.3. Regulatory Pathways and Market Access

The translation of diagnostic technologies from prototypes to clinical practice is governed by regulatory frameworks. As early as 2007, developers of EIS systems recognized that receiving regulatory approval was as critical as developing prototypes [56]. In the United States, devices with diagnostic algorithms are evaluated based on their risk profiles. For instance, the aforementioned Nevisense was classified as a high-risk Class III device, requiring a strict premarket approval (PMA) pathway [57,58]. Nevisense received FDA PMA in 2017, but its authorization was limited to use by dermatologists [16].

Later, in 2024, DermaSensor (DermaSensor Inc., Miami, FL, USA) received approval via the de novo pathway as a Class II (low-to-moderate risk) device [16]. Unlike Nevisense, DermaSensor is an AI-enabled elastic scattering spectroscopy device explicitly indicated for PCPs, which lowers the barrier for the clinical use of objective diagnostic tools [16]. The regulatory concern mainly arises from the “black box” nature of the algorithms in these dermatology devices, making it challenging to evaluate whether the device outputs follow established clinical standards [58].

## 4. Clinical Efficacy and Validation in Skin Cancer Diagnosis

### 4.1. Evidence for Improved Diagnostic Accuracy and Clinical Utility

#### 4.1.1. Foundational Evidence from Multicenter Clinical Trials and Algorithmic Trade-Offs

The potential of EIS in the diagnosis of skin cancer has been validated by numerous rigorous and multicenter trials [59]. A comprehensive summary of the clinical performance, including sensitivity and specificity, of various EIS systems is presented in Table 1.

Malvehy et al. employed the Nevisense EIS system in the detection of melanoma across 22 research centers and 1951 participants, demonstrating sensitivity of 96.6% and specificity of 34.4% [55]. Similarly, Mohr et al. analyzed 1300 lesions to optimize the classification algorithms of an EIS system, reaching high sensitivity of 99.4% at the expense of low specificity (24.5%) [60]. This low specificity indicates that some benign lesions with similar electrical properties to cancerous tissues, such as actinic keratoses, could be classified as malignancies [55]. This sensitivity-oriented design helps to reduce missed melanomas, although the associated false positive burden should also be considered [55].

#### 4.1.2. Improving Biopsy Decisions Beyond Dermoscopy

One of the most important contributions of EIS is assisting doctors’ decisions regarding whether to perform biopsies on skin lesions [74]. This approach has been validated in various studies, particularly in addressing the limitations of dermoscopy, which can complicate the diagnosis of benign pigmented skin lesions (PSLs) [21]. Zakria et al. conducted a comprehensive study involving 151 dermatologists, demonstrating that dermoscopy improved the correct biopsy decision rate in MM from 56.2% for visual inspection to 78.5%. However, it also decreased the accuracy for benign PSLs to 48.0%, which was even lower than that of sole visual inspection (59.7%) [21]. With the integration of EIS data, the correct biopsy rate for benign lesions could be increased to 58.7%, and the accuracy for MM was improved to 86.2% [21]. Despite its effectiveness, clinicians chose not to accept the EIS suggestion in 16% of biopsy decisions, further demonstrating that EIS works better as a complementary tool rather than a definitive diagnostic method [21].

Interestingly, the clinical effectiveness of EIS could be influenced by regional healthcare environments. Zakria et al. performed a comparative study involving 231 US and 151 German dermatologists, demonstrating that, although EIS improves biopsy decisions in general, the specific tendency is different [74]. American dermatologists had a significantly higher rate of correct biopsy decisions for MM (91.1% vs. 86.2% with EIS) and severe dysplastic nevi (79.1% vs. 68.1% with EIS) but had a significantly lower rate of correct biopsy decisions for benign PSLs (41.5% vs. 58.7% with EIS), suggesting that they had a lower overall threshold for performing biopsies on PSLs than their German counterparts. This difference is largely attributed to the lower biopsy threshold among US physicians, since there is a higher malpractice litigation risk (estimated at 5–6% annually) compared to Germany [74]. These findings reveal that EIS not only provides objective biophysical data but also helps to standardize clinical workflows across different healthcare systems.

#### 4.1.3. Enhancing Diagnostic Efficiency by Reducing Unnecessary Biopsies

Beyond improving diagnostic accuracy, EIS technology could improve diagnostic efficiency through optimizing clinical processes [8]. In dermatological scenarios, many biopsies are performed on benign lesions to avoid missing melanomas, which is not only a waste of medical resources but also creates an unnecessary burden for patients [9]. EIS technology could effectively lower the rate of unnecessary biopsies by providing non-invasive and objective information about skin tissues [73].

The capacity of EIS to refine biopsy decisions has been validated in clinical practice. For instance, a retrospective analysis of 909 suspicious lesions in a private dermatology practice demonstrated that integrating the Nevisense EIS system with visual and dermoscopic inspection reduced the number needed to excise (NNE) from 17.5 to 7.8 [54]. The reason that doctors are empowered to rule out malignancy lies in the high negative predictive value (NPV) of EIS technology. This metric indicates that, when EIS data show that the lesion is benign, the conclusion has high credibility. Specifically, lesions receiving a negative EIS score (0–3) exhibited an NPV of 98.9%, providing clinicians with the confidence to reduce biopsies [54,75].

The efficiency improvement is not only evident in reducing surgeries but also in the triaging of patients (Figure 4B) for short-term sequential digital dermoscopy imaging (SDDI) [70]. SDDI is a standard protocol for monitoring suspicious lesions, but it requires delayed diagnosis and repeated patient visits. A prospective study by Rocha et al. integrated EIS measurements into the SDDI workflow, establishing a risk-stratified protocol: lesions with an EIS score of 0–3 were discharged from monitoring, those with scores of 4–6 underwent standard 3-month SDDI, and those with scores of 7 or higher were immediately excised [70]. This approach reduced the need for SDDI follow-ups by 46.9%, and the sensitivity for the detection of MM was maintained at 100% [70].

Overall, the current clinical evidence supports EIS as an adjunct that can improve biopsy decisions and reduce the number needed to excise [54]. The 96.6% sensitivity reported in the multicenter Nevisense trial was accompanied by 34.4% specificity [55], and another large study reported 99.4% sensitivity at 24.5% specificity [60]. Taken together, these operating points favor sensitivity over specificity, and EIS is therefore best interpreted alongside clinical and dermoscopic assessment [59]. These observations also motivate future evaluations of cost-effectiveness, patient-reported outcomes, and workflow impacts.

### 4.2. Diagnostic Performance for Specific Cutaneous Malignancies

#### 4.2.1. Performance in Malignant Melanoma (MM) Detection

EIS typically shows high overall sensitivity in detecting melanoma, indicating its ability to avoid the missed identification of lesions. However, its performance is highly dependent on the tumor’s developmental stage.

In particular, the biophysical complexity of melanoma introduces specific diagnostic challenges, particularly concerning the tumor thickness, central necrosis, and the integrity of the stratum corneum (SC). At the tissue level, the electrical response of a melanoma is dynamic. A study by Glickman et al., utilizing a TransScan prototype (operating between 100 Hz and 100 kHz at 2.5 V), demonstrated that the normalized conductivity at 2 kHz significantly increases as the tumor proliferates [64]. As tumors grow larger, central necrosis often occurs, which decreases the local conductivity [64]. This non-monotonic behavior directly impacts the diagnostic accuracy based on the tumor depth and size. Moreover, although EIS achieves 100% sensitivity for melanomas thicker than 1.0 mm, its sensitivity drops to 89% for those thinner than 1.0 mm [62]. Moreover, while some studies report 100% accuracy for melanoma detection [54], such results must be reconsidered due to the small absolute number of melanomas in these cases (e.g., only three melanomas out of 909 lesions) [54].

Despite its high sensitivity for more advanced lesions, the diagnosis of early-stage melanoma, especially melanoma in situ (MIS), remains a typical challenge for EIS [35,61]. In a study involving 19 high-risk patients, EIS identified 15 of them to be melanomas, while it misclassified four cases as benign, showing sensitivity of only 78.9%. All four missed cases were small-diameter or in situ lesions [61]. The reason lies in the SC barrier. At a measurement frequency of 1 kHz, invasive melanoma exhibits 50% lower electrical resistance (dropping from approximately 80.3 kΩ in healthy skin to 35.1 kΩ) due to the destruction of the SC barrier [35].

Melanoma is heterogeneous in both anatomical presentation and growth pattern. Acral lentiginous melanoma typically involves acral or nail-associated sites, and melanoma in situ is confined to the epidermis. Nodular and superficial spreading melanoma also have different growth patterns [76]. This heterogeneity is relevant to EIS because existing evidence indicates reduced sensitivity for some small or in situ lesions [61]. Accordingly, future EIS studies should report its performance by melanoma subtype and anatomical site.

#### 4.2.2. Performance in Non-Melanoma Skin Cancer (NMSC) Detection

Non-melanoma skin carcinomas (NMSCs), primarily comprising basal cell carcinoma (BCC) and squamous cell carcinoma (SCC), are prevalent skin tumors in clinical practice [47,65]. By employing a depth-selective approach across a frequency range of 1 to 1000 kHz, the resulting electrical impedance spectra (both magnitude and phase) can effectively capture the distinct dielectric properties of a BCC compared to a benign nevus and adjacent healthy reference skin across multiple depth settings [47]. As a result, EIS technology shows great potential in detecting NMSCs, with high sensitivity of 98.4% [65], 94.2% [66], and even 100% [47] in recent research.

Despite this high sensitivity, the evaluation of BCC with EIS faces specific hardware and subtype challenges. A critical hardware challenge arises from the rigid nature of the electrodes. The identification of BCC relies on several metrics, including the amplitude index (MIX), phase index (PIX), and imaginary part index (IMIX), which are lower in BCC lesions than in benign lesions or normal skin [77]. However, conventional probes utilizing 10-mm-diameter electrodes often exceed the size of early BCC lesions (typically 2–15 mm), and the coverage of adjacent healthy skin with high impedance can mask the difference between BCC lesions and normal skin [77].

To overcome these hardware limitations, researchers have developed advanced analytical methods to achieve more accurate diagnoses [72]. For instance, artificial neural networks have shown great potential in improving the diagnostic performance of EIS in BCC. A model achieved 100% accuracy on the test set after preprocessing the data while accounting for the lesion size and surrounding normal skin impedance [72]. Additionally, it remains difficult to differentiate BCC subtypes, such as nodular BCC (NBCC) and superficial BCC (SBCC). It is reported that the baseline impedance varies significantly across body locations (e.g., the face for NBCC versus the trunk for SBCC). Therefore, it is suggested to measure the impedance of adjacent healthy skin as a reference, rather than directly comparing the absolute impedance values [78].

In the diagnosis of SCC, doctors usually face two challenges: determining the depth of the tumor and distinguishing SCC from inflammatory seborrheic keratosis (SK). Histologically, these lesions are often difficult to distinguish through visual inspection. Invasive SCC has tumor islands that fill the dermis while the epidermis is filled with atypical cells, but these do not invade into the dermis in SCC. In the case of inflamed SK, epidermal hyperplasia occurs, with perivascular and interstitial inflammatory cell infiltrates in the superficial dermis [67]. EIS can detect the electrical differences between these three cases. An algorithm was developed to distinguish SCC in situ from inflamed SK, with high accuracy of 95.8%, sensitivity of 94.6%, and specificity of 96.9% [67]. However, when this algorithm was employed to distinguish between normal skin and SCC, the specificity dropped to 51.2%, with high sensitivity of 90.2% [67]. This indicates a high risk of false positives.

EIS technology has been extended to the detection of oral squamous cell carcinoma (OSCC), demonstrating that cancerous tissues in OSCC have lower resistance and reactance than healthy tissues (*p* < 0.0001) [29,53]. Sarode et al. developed a four-electrode disposable probe and applied a low 200 mV amplitude to address the interference of contact impedance [29]. It was revealed that the greatest distinctions between OSCC and healthy tissue were observed at 20 Hz and 50 kHz, and these low frequencies reflect the electrical response in extracellular ionic environments [29]. Furthermore, the impedance of OSCC declines with the severity of the disease. At a frequency of 20 Hz, the mean impedance decreased from 4881 ± 262.5 Ω in stage I to 4376 ± 121.3 Ω in stage III and 4500 ± 181.6 Ω in stage IV [29]. Similarly, poorly differentiated tumors present lower impedance (4347 ± 76.12 Ω) compared to well-differentiated ones (4557 ± 260.8 Ω) (*p* = 0.0004) [29]. This reduction in impedance reveals the growing damage to the tissue structure with tumor growth.

#### 4.2.3. Evaluating Precancerous Lesions Including Actinic Keratosis

EIS could also be employed to evaluate precancerous lesions. For instance, EIS technology has been adopted to detect actinic keratosis (AK) and its associated field cancerization (FC) [68]. Berger et al. used the Nevisense system to measure 55 patients with multiple AKs, achieving sensitivity of 98.0% for AKs and 82.0% for FC lesions [68]. However, it was revealed that the EIS values did not decrease even after four weeks of therapy with imiquimod or ingenol mebutate. This could have been due to the small sample size, insufficient therapy effectiveness, low patient compliance, or a not completely subsided inflammatory reaction due to topical therapy at the time of follow-up [68].

To address the cost and invasiveness of current commercial systems, the DermaSense prototype employs a non-invasive scanning head with eight spherical stainless-steel electrodes arranged in a circular pattern for the measurement of AKs [18]. Operating at a constant voltage of 1.68 V across a frequency range of 100 Hz to 14 kHz, it evaluated the electrical response of the lesion without penetrating the highly resistive stratum corneum [18]. However, it is still at an early stage of development, requiring improvement and optimization.

#### 4.2.4. Potential for Intraoperative Margin Assessment

At present, EIS technology is widely considered as an in vivo diagnostic tool, while scientists are exploring its use in surgical procedures. A specific example is the employment of EIS in Mohs micrographic surgery (MMS) to evaluate whether the surgical margins are clean or not [69,79]. The determination of tumor boundaries is a great challenge in MMS that significantly increases the surgical time, and ex vivo EIS technology could reduce the number of repeated resections and thus reduce the duration of surgery [79].

The measurement relies on the distinct water and electrolyte compositions of cellular components in cancerous epithelial cells compared to normal adjacent tissues [69]. In a study analyzing 151 MMS specimens of non-melanoma skin cancer (NMSC) using the MarginScan device(NovaScan, Inc., Chicago, IL, USA), researchers measured the bioimpedance of the excised tissues and fitted the data to a Cole–Cole function curve to extract specific relaxation frequencies [69]. The system successfully differentiated malignant margins from healthy tissue, with sensitivity of 95.6% and specificity of 99.1% [69].

## 5. Advanced Frontiers in EIS Diagnostics

### 5.1. Integration with Artificial Intelligence and Machine Learning

As mentioned above, EIS has been clinically validated as an effective diagnostic tool, while research is ongoing to further enhance its performance and clinical utility. There are two major directions for its development, namely the introduction of artificial intelligence and the integration of EIS with other non-invasive technologies.

The introduction of artificial intelligence is not a recent innovation. Early researchers recognized that EIS data were often too complex for direct human analysis [72], which naturally led to the adoption of artificial neural networks (ANNs) as analytical tools. For instance, an early study by Dua et al. utilized a feed-forward neural network to classify basal cell carcinoma (BCC) and benign lesions using impedance measured across 31 frequencies from 1 kHz to 1 MHz at five virtual depths [72]. Although the network achieved classification accuracy of 100%, the study had a small sample size of only 18 BCC and 16 benign lesions.

Other deep learning architectures besides basic ANNs have also been developed. For example, the DermaSense prototype system evaluated various ANN architectures to distinguish melanocytic nevi from normal skin [5]. The study found that an architecture employing rectified linear unit (ReLU) activation achieved accuracy and sensitivity of 77.4% and 94.7%, respectively. Moreover, Gessert et al. designed a recurrent neural network employing gated recurrent units (GRUs) with a state-max-pooling mechanism [71]. Furthermore, this model was integrated with convolutional neural networks (CNNs) to achieve multimodal fusion. The optimized model achieved high sensitivity of 98% and specificity of 53.7%, making it superior to models relying on EIS (34.7% specificity) or dermoscopy alone (34.4% specificity) [71].

Building on the joint EIS–dermoscopy deep learning model reported by Gessert et al. [71], future work could examine transformer-based or foundation-model architectures for multimodal integration and federated learning for multicenter development. Clinical translation would require the validation procedures emphasized in reviews of approved dermatology AI systems [57] and the regulatory assessment discussed for FDA-cleared dermatology software [58].

### 5.2. Multimodal Fusion and Comparison with Competing Non-Invasive Technologies

**Figure 5:**
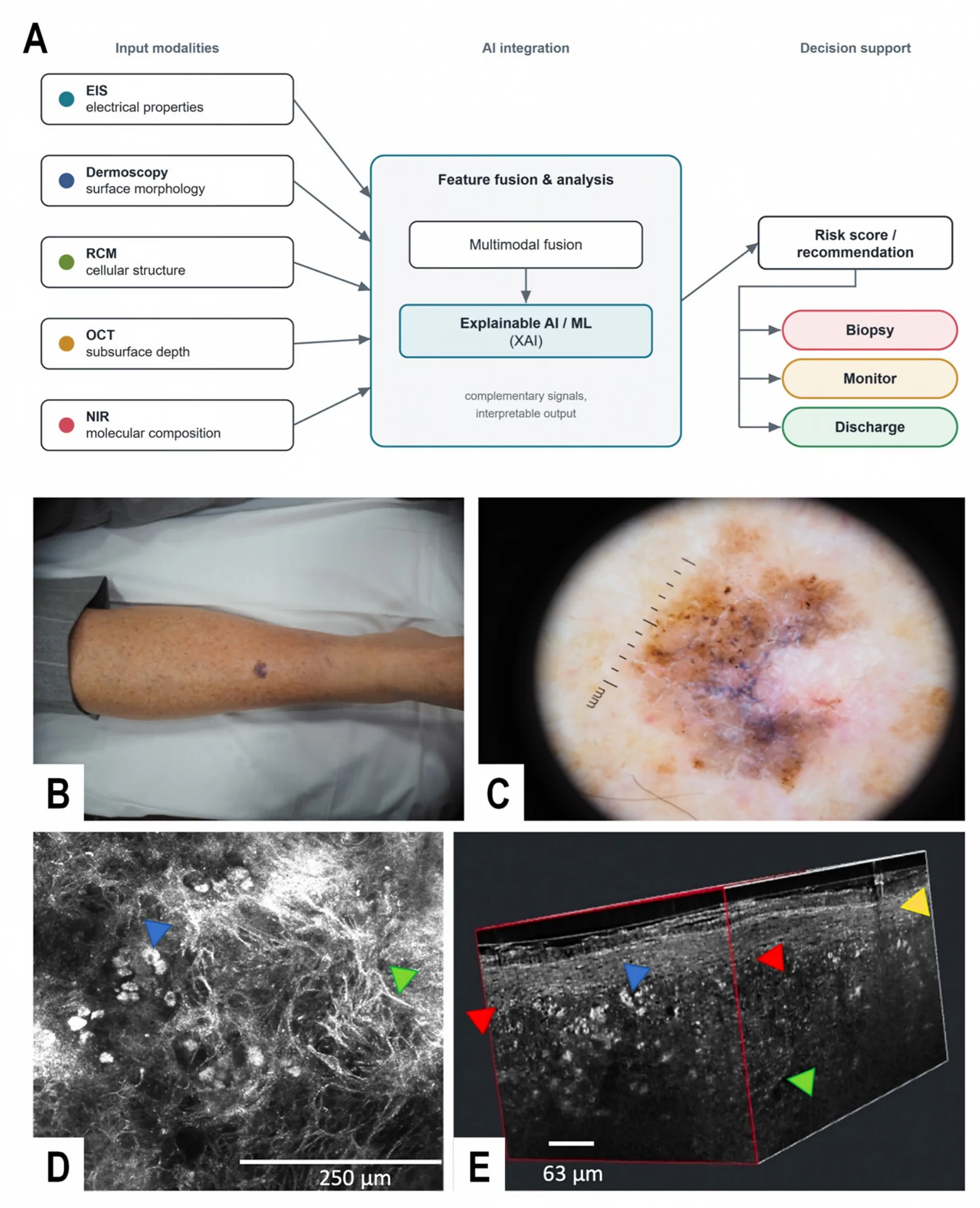
(**A**) Proposed framework: complementary non-invasive modalities—EIS (electrical properties), dermoscopy (surface morphology), reflectance confocal microscopy (RCM; cellular structure), optical coherence tomography (OCT; subsurface depth), and near-infrared spectroscopy (NIR; molecular composition)—are integrated by explainable machine learning models (XAI) into a single interpretable risk estimate that guides biopsy, monitoring, or discharge. Melanoma in three different imaging techniques: (**B**) clinical picture; (**C**) dermoscopic image; (**D**) reflectance confocal microscopy: cellular atypia (blue arrow) and dendritic cells (green arrow); (**E**) 3D block of LC-OCT: irregular epidermis (yellow arrow), disruption of the dermoepidermal junction (red arrow), and atypical nests with atypical (blue arrow) and dendritic cells (green arrow). Reproduced from Ref. [10], 2022, MDPI; licensed under CC BY 4.0.

Beyond introducing AI to optimize the performance of EIS, researchers have been working on the integration of EIS with other non-invasive technologies, including digital dermoscopy, reflectance confocal microscopy (RCM), optical coherence tomography (OCT), and multispectral imaging (Figure 5A) [12,14,80,81]. A comparison of the characteristics, clinical roles, and diagnostic performance of these non-invasive technologies is summarized in Table 2.

These distinct non-invasive technologies could be used to assess different characteristics of skin lesions and provide complementary information (Figure 5B) [10]. For instance, RCM could provide high-resolution structural visualization [12,14], and dermoscopy could output information about the surface morphology. In parallel, EIS and multispectral analysis help to evaluate the skin’s response to different microcurrents or wavelengths [14]. Through the comprehensive evaluation of the above information, clinicians could achieve more precise cancer diagnoses and reduce the number of unnecessary biopsies on benign lesions [7,14,81]. A recent dermatological guideline also suggests that clinicians should perform diagnosis through a combination of these methods [12].

As mentioned above, the integration of EIS with digital dermoscopy has proven effective in optimizing clinical workflows, in which EIS can reduce unnecessary SDDI follow-ups [70]. Beyond the optimization of clinical workflows, Har-Shai et al. combined EIS data with the analysis of dermoscopic images, demonstrating a detection rate of 100% when evaluating melanomas [63]. The study utilized the TransScan TS2000M system (TransScan Medical Ltd., Migdal Ha’Emek, Israel). They integrated automated morphological parameters from digital images with conductivity maps, enabling a comprehensive evaluation of early invasive lesions.

The integration of EIS with other high-resolution imaging technologies like RCM and OCT is also being developed to address the deficiencies of EIS [12]. For instance, EIS could acquire false negatives in the detection of early-stage or in situ melanomas (MIS) that have not disrupted the SC barrier. Therefore, the electrodes cannot assess cancerous tissues under the SC barrier [35,61]. In these cases, RCM can directly reveal underlying cancerous features, such as atypical cobblestone patterns, non-edged papillae, and dendritic pagetoid infiltration [61], providing complementary information for EIS.

The combination of near-infrared (NIR) spectroscopy with EIS has shown promising potential. An analysis of combined EIS-NIR data showed 100% sensitivity and 100% specificity in distinguishing cancerous and precancerous skin tumors from benign lesions [17]. However, the same combined EIS-NIR method demonstrated sensitivity of 83% and specificity of 95% in another case of 50 lesions including 19 dysplastic nevi [73]. This reveals that distinguishing between atypical dysplastic nevi and early melanoma is still challenging.

Beyond dermoscopy, RCM, OCT, and NIR, several technologies address different steps of the diagnostic pathway, although awareness and adoption of EIS in routine practice remain limited [82], and some consensus recommendations have not yet defined its role [83]. In a prospective study, adding high-frequency ultrasound to clinical examination improved the diagnosis and management of equivocal skin lesions [84], whereas a two-gene pigmented-lesion assay used adhesive-patch RNA to enable the non-invasive molecular classification of suspicious melanocytic lesions [85]. High-frequency ultrasound has also been evaluated perioperatively for margin assessment during micrographic-controlled surgery [86].

Hyperspectral imaging adds another optical route. Lin et al. used hyperspectral image enhancement with machine learning classification to distinguish actinic keratosis, basal cell carcinoma, and seborrheic keratosis [87]. In a multimodal framework, such optical information could complement EIS-derived electrical features, but this remains a future research proposition rather than a clinically validated combination.

## 6. Conclusions and Perspectives

This review covers the development of EIS from its principles to its clinical applications in the diagnosis of skin cancer. EIS could provide a measurable dielectric contrast between cancerous and healthy tissues, which serves as objective and quantitative data to assist dermoscopy diagnosis and to address the high rate of unnecessary biopsies. The aspects of hardware evolution, clinical validation, and emerging directions such as AI-driven integration have been discussed. These findings support EIS as a valuable component of multimodal skin lesion assessment.

From a clinical perspective, the utility of EIS has been supported by large-scale, multicenter trials, showing its value in decision support. When interpreted together with clinical and dermoscopic findings, EIS can maintain high sensitivity while providing objective information that supports biopsy decisions. Reported reductions in the number needed to biopsy are encouraging for patients and healthcare resources, while future studies may further evaluate cost-effectiveness and patient-reported outcomes. A further obstacle to the widespread use of EIS is the gap between reported clinical validation and clinical practice. A cross-sectional survey across nine European countries found that only 6.9% of plastic surgeons were aware of EIS, and none of them had employed EIS in their clinical practice [82]. Moreover, a consensus panel on the detection of melanoma did not define the role of EIS as an evaluation tool for pigmented lesions [83]. Future efforts should include technological innovation, user education, clinical guidelines, and suggested workflows that integrate EIS with existing clinical processes.

EIS has shown high sensitivity in avoiding missed cancerous lesions. However, it has low specificity, resulting in an increasing number of false positive results. Improving the specificity of EIS without compromising its sensitivity remains one of the major challenges, which requires both the development of AI technologies and integration with other non-invasive technologies. At the technical level, an important development direction is to improve the diagnostic accuracy for early and in situ lesions. Although EIS systems have been proven effective in identifying typical cancerous lesions, they need further improvement to distinguish lesions with smaller electrical differences, including melanoma in situ from invasive melanoma and different types of BCC. Research efforts are also needed in terms of both hardware and software. In the field of hardware, sensor arrays with higher spatial resolutions are needed to distinguish subtle tissue differences. In the field of software, signal processing techniques and machine learning algorithms should be optimized to analyze these minor differences.

Besides the persisting demand for higher diagnostic accuracy, the integration of EIS with AI faces several challenges, including diagnosis biases and explainability. The diagnosis biases are mainly due to the lack of a comprehensive training datasets that include various skin and lesion types. Moreover, these AI-driven algorithms often share a black-box nature that hinders their acceptance among clinicians. If the reasons behind AI recommendations remain unknown, clinicians will be hesitant to form a diagnosis following the use of these algorithms. Future research should focus on developing explainable AI (XAI) frameworks that provide interpretable recommendations, and these frameworks should be validated through large-scale trials to ensure reliability. Future point-of-care systems may integrate flexible electrodes, smartphone-linked acquisition, cloud-assisted analysis, personalized risk assessment, teledermatology, and longitudinal monitoring, with appropriate attention to privacy and regulatory requirements.

The most compelling direction for EIS may be not as a standalone tool but as one component within a multimodal diagnostic framework. For instance, combining its electrical measurements with dermoscopic images could provide more comprehensive information for clinicians that a single technology cannot provide. In this multimodal framework, AI could act as an inexhaustible tool to analyze data from different diagnostic technologies and provide explainable recommendations. If this integration can be implemented successfully in practice, it could rebuild clinical workflows for skin cancer diagnosis to provide more precise and effective outcomes.

## Figures and Tables

**Table 1:** Clinical performance and critical comparison of EIS systems in skin cancer diagnosis.

Reference	Diagnostic Scenario	EIS System/Method	Sample Size	Sensitivity (%)	Specificity (%)
[55]	Melanoma vs. Benign	Nevisense (microinvasive)	1943 lesions	96.6	34.4
[60]	Melanoma and NMSC vs. Benign	SciBase III	1300 lesions	98.1–99.4	23.6–24.5
[54]	Melanoma and NMSC	Nevisense	909 lesions	100.0	N/A
[61]	Changing Lesions	Nevisense	68 lesions	78.9	22.0
[62]	Melanoma vs. Benign	SciBase II prototype	210 lesions	95.0	49.0
[63]	Melanoma vs. Benign	TransScan TS2000M	449 lesions	91.0	64.0
[41]	MM and BCC vs. Benign	SciBase II	140 lesions	92.0 (MM), 96.0 (BCC)	80.0 (MM), 86.0 (BCC)
[64]	Melanoma and Dysplastic vs. Benign	TransScan prototype	178 lesions	92.0 (MM), 91.0 (dysplastic)	67.0 (MM), 59.0 (dysplastic)
[65]	NMSC vs. Benign	Nevisense	1712 lesions	98.4	N/A
[66]	NMSC vs. Benign	Nevisense	200 lesions	94.2	41.9
[47]	NMSC vs. Benign	SciBase II	611 lesions	100.0	87.0
[67]	SCC vs. Inflamed SK	Electrical impedance dermography	35 subjects	94.6	96.9
[29]	OSCC vs. Healthy Mucosa	Four-electrode probe	100 subjects	N/A	N/A
[68]	Actinic Keratosis (AK) and Field Cancerization (FC)	Nevisense	55 patients	98.0 (AK), 82.0 (FC)	N/A
[69]	MMS Intraoperative Margin Assessment	MarginScan (Ex vivo)	151 specimens	95.6	99.1
[70]	Short-Term SDDI Monitoring	Nevisense + SDDI	160 lesions	100.0	69.5
[71]	Melanoma vs. Benign	Nevisense + dermoscopy (deep learning)	988 lesions	98.0	53.7
[5]	Melanocytic Nevi vs. Normal Skin	DermaSense prototype + ANN	155 lesions	94.7	50.0
[72]	BCC vs. Benign	SciBase prototype + ANN	34 lesions	N/A	N/A
[17]	Malignant/Premalignant vs. Benign	EIS + NIR	Primary healthcare center	100.0	100.0
[73]	Melanoma vs. Dysplastic/Benign	EIS + NIR	50 lesions	83.0	95.0

**Table 2:** Comparison of characteristics of EIS and selected non-invasive technologies for skin cancer assessment.

Technique	Device Format/Cost	Typical Clinical Role	Depth/Information	Representative Evidence and Limitations
EIS	Mobile prototype and cost-efficient research prototype reported [18]; commercial cost was not assessed	Adjunctive assessment of lesions selected for melanoma evaluation [55]	Electrical current penetration up to approximately 2.5 mm [13]	Melanoma sensitivity was 96.6% [55] and 99.4% with algorithm 2 [60], with corresponding specificities of 34.4% [55] and 24.5% [60]. Limitations include low specificity [55] and lesion coverage or anatomical site restrictions [13].
Dermoscopy	Handheld and digital systems [12]; commercial cost was not assessed	First-line morphological assessment [10]	Epidermis and upper dermis [10]	For BCC, pooled sensitivity and specificity were 91.2% and 95%, respectively [12]. Accuracy depends on operator experience and training [10].
RCM	Fixed wide-probe and handheld systems with high equipment cost [10]	Second-level evaluation of dermoscopically equivocal lesions [10]	Cellular-resolution imaging to 250–300 μm [10]	For melanoma, sensitivity and specificity of 93% and 76%, respectively, were reported [10]. Limitations include imaging depth and extensive training requirements [10].
OCT	Clinical imaging systems with high equipment cost [10]	Structural assessment, mainly for NMSC/BCC [10]	Architectural imaging to approximately 2 mm [10]	For BCC, sensitivity and specificity of 89.3% and 60.3%, respectively, were reported [13]. Cellular detail is lower than in RCM, and training is required [10].

## Data Availability

No new data were generated or analyzed in this study. Data sharing is not applicable to this article.

## Abbreviations

The following abbreviations are used in this manuscript:

EIS	Electrical Impedance Spectroscopy
MM	(Malignant/Cutaneous) Melanoma
MIS	Melanoma In Situ
BCC	Basal Cell Carcinoma
NBCC/SBCC	Nodular/Superficial Basal Cell Carcinoma
SCC	Squamous Cell Carcinoma
OSCC	Oral Squamous Cell Carcinoma
NMSC	Non-Melanoma Skin Cancer
ADC	Adenocarcinoma
H&NC	Head and Neck Squamous Cell Carcinoma
AK	Actinic Keratosis
FC	Field Cancerization
SK	Seborrheic Keratosis
PSL	Pigmented Skin Lesion
SC	Stratum Corneum
RCM	Reflectance Confocal Microscopy
OCT	Optical Coherence Tomography
NIR	Near-Infrared (Spectroscopy)
SDDI	Sequential Digital Dermoscopy
FEM	Finite Element Method
GET	Graphene Electronic Tattoo
SoC	System-on-Chip
SNR	Signal-to-Noise Ratio
AI	Artificial Intelligence
ANN	Artificial Neural Network
CNN	Convolutional Neural Network
GRU	Gated Recurrent Unit
ReLU	Rectified Linear Unit
XAI	Explainable Artificial Intelligence
NPV	Negative Predictive Value
